# Factors affecting carbon dioxide emissions for sustainable development goals – New insights into six asian developed countries

**DOI:** 10.1016/j.heliyon.2024.e39943

**Published:** 2024-10-30

**Authors:** Pham Xuan Hoa, Vu Ngoc Xuan, Nguyen Thi Phuong Thu

**Affiliations:** a Department of Public Finance, School of Banking and Finance, National Economics University, Viet Nam; b Faculty of Economics, College of Economics and Public Management, National Economics University, Viet Nam; c Faculty of Planning and Development, College of Economics and Public Management, National Economics University, Viet Nam

**Keywords:** Ecological contamination, (EC), Sustainable energy consumption, (REC), Foreign direct investment, (Cross-border investment- FDI), Gross domestic product, (GDP)

## Abstract

The worldwide economic scene faces the dual challenges of rising sea levels and escalating carbon dioxide (CO2) emissions. Environmental contamination impedes sustainable growth, increasing the demand for sustainable energy resources as a fundamental aspect of sustainable economies. The present research uses an analytical model to examine the elements influencing carbon dioxide output in six economically developed Asian nations, intending to assist in realizing the UNSDG- United Nations' Sustainable Development Goals. The manuscript applies the fixed effect model- FEM and the random effects approach- REM method. The research contributes to the science of the environment, climate research, and long-term environmental growth. Data for this investigation were sourced from the World Bank, covering the period from 2000 to 2020, about Hong Kong, Israel, Japan, Korea, Singapore, and China. This paper investigates the nexus between power usage (ECO), fossil fuel use (FFU), sustainable energy adoption (REC), foreign direct investment (FDI), imports, exports, economic development, population, and ecological contamination across six developed Asian countries. The study uses panel data regression analysis to examine how these factors influence carbon dioxide output, aiming to provide insights for long-term environmental growth policies. Key findings highlight the considerable effect of energy consumption patterns and economic activities on pollution levels, emphasizing the need for enhancements in energy conservation, a shift towards sustainable energy sources, and practical rules protecting the environment to mitigate environmental degradation. The empirical findings indicate that various factors, such as power usage, fossil fuel-based energy, sustainable energy usage, foreign direct investment (FDI), import and export activities, economic development, and population size, impact ecological contamination within these six nations. Specifically, FDI, economic development, and green energy influenced environmental degradation negatively. Conversely, factors such as power usage, energy from fossil sources usage, imports, exports, and population size correlate positively with ecological contamination. The research consequently advocates for developing green, circular, and sustainable economic frameworks within these six developed Asian countries in the foreseeable future.

## 1 Introduction

The main objective of this research is to explore the complex interactions between power usage, fossil fuel use, sustainable energy adoption, cross-border investment (FDI), trade (imports and exports), economic development, population, and ecological contamination in six advanced Asian nations: Japan, South Korea, Singapore, Hong Kong, China, and Israel. Using panel data from 2000 to 2020, the study seeks to Identify Key Drivers of Environmental Pollution and pinpoint the significant contributors to pollution, especially carbon dioxide output, within the context of these developed Asian economies. Assess Energy Consumption Impact: Investigate how electricity uses and the balance of fossil fuels and sustainable energy affect pollution levels. Analyze the Role of Economic Activities: Examine the influence of FDI, trade, economic development, and population on ecological contamination. Provide Policy Recommendations: Offer data-driven recommendations for policies to help these countries align economic development with ecological sustainability [[1], [2], [3]].

This paper seeks to examine the intricate relationships between power usage, fossil fuel usage, sustainable energy adoption, cross-border investment (FDI), trade (both imports and exports), economic development, population, and ecological contamination across six developed Asian countries: Japan, South Korea, Singapore, Hong Kong, China, and Israel. By analyzing panel data from 2000 to 2020, the study strives to Identify Major Contributors to Environmental Pollution and highlight the key factors, particularly carbon dioxide output, that drive pollution in these economies. Evaluate the Impact of Energy Use: Assess how power usage, fossil fuels, and sustainable energy use influence pollution levels. Investigate the Effects of Economic Activities: Explore how FDI, trade, economic development, and population impact ecological contamination. Provide Policy Insights: Present supported by empirical evidence recommendations to support the balance between economic development and ecological sustainability in these countries [1,4,5].

Sustainable Development Goals (SDGs)- The findings support the achievement of several United Nations Sustainable Development Goals (SDGs), particularly.

• SDG 7 (Affordable and Clean Energy): Emphasizing the importance of transitioning to sustainable energy sources to reduce pollution.
• SDG 13 (Climate Action): Providing evidence on the drivers of carbon dioxide output to inform climate action strategies.
• SDG 8 (Decent Work and Economic Growth) and SDG 9 (Industry, Innovation, and Infrastructure) Highlight the need for sustainable economic development and innovation in energy and environmental technologies.

Methodological Contributions: This study adopts a rigorous methodological framework using advanced panel data regression techniques and conducting thorough diagnostic tests. It employs fixed-effects models to account for unobserved heterogeneity, and the inclusion of various diagnostic tests strengthens the reliability of the findings. This approach can be replicated in similar studies across other regions or countries. Future Research Directions: The study highlights several potential areas for future exploration. Expanding Geographic Coverage: Including more countries or regions could provide a more comprehensive view of the global relationship between economic activities and ecological contamination. Longer Time Frames: Extending the analysis to cover longer periods, potentially incorporating historical data from the 2000s or earlier, could reveal long-term trends and the effects of significant policy shifts. Advanced Econometric Methods: More sophisticated techniques, such as instrumental variable approaches or dynamic panel data models, could address endogeneity issues and yield more robust conclusions. In summary, this paper seeks to enhance the understanding of the complex relationship between economic activities, energy consumption, and ecological contamination in developed Asian countries. The findings stress the need for informed policymaking to promote long-term environmental growth and ecological preservation [[6], [7], [8]].

Understanding the nexus between economic activities, energy consumption, and ecological contamination remains crucial in global efforts toward long-term environmental growth. This study focuses on six developed Asian countries—Japan, South Korea, Singapore, Hong Kong, China, and Israel—from 2000 to 2020. The primary objective is to analyze the relationships among power usage, fossil fuel use, sustainable energy adoption, cross-border investment (FDI), imports, exports, economic development, population, and carbon dioxide output. Research Objective- The overarching goal is to elucidate how these factors contribute to ecological contamination in developed Asian economies. By investigating these relationships, the study seeks to provide empirical evidence and insights that can inform policy interventions to achieve long-term environmental growth goals [1,9].

Research Gap- Previous studies have explored the relationship between economic development, energy consumption, and environmental outcomes. However, gaps persist, particularly in the context of Developed Asian Economies: Existing literature predominantly focuses on global trends or specific regional contexts, often overlooking the unique characteristics and challenges faced by developed Asian countries. Integrated Analysis: Many studies examine isolated factors such as energy consumption or economic development without fully integrating the complex interactions among variables like FDI, trade, population dynamics, and their collective impact on ecological contamination. Policy Relevance: While theoretical frameworks exist, there is a need for empirical studies that provide actionable insights for policymakers in developing effective strategies to mitigate environmental degradation while fostering economic development. Contribution- This study seeks to narrow these gaps by conducting a comprehensive analysis using panel data regression techniques. By synthesizing data from reputable international sources and applying rigorous statistical methods, the research offers nuanced insights into the interplay between economic activities, energy use, and ecological sustainability in developed Asian contexts. The findings are supported by empirical evidence policymaking that promotes long-term environmental growth practices tailored to these economies' specific challenges and opportunities [1,10,11].

Understanding the complex relationships between economic activities, energy consumption, and ecological contamination is crucial for long-term environmental growth, particularly in the context of developed Asian countries. This study focuses on six specific nations—Japan, South Korea, Singapore, Hong Kong, China, and Israel—from 2000 to 2020. The rationale behind selecting these countries is multifaceted and rooted in their unique economic, environmental, and geopolitical characteristics, which make them compelling subjects of analysis. Economic Significance- Technological Advancement: These six countries are renowned for their significant advancements in technology and innovation. They represent a blend of highly industrialized economies with robust manufacturing sectors and a strong emphasis on technological advancement and research and development (R&D) capabilities. This technological prowess influences their energy consumption patterns, environmental policies, and economic development trajectories, making them critical case studies for understanding the nexus between economic development and ecological sustainability [12,13].

Trade and Investment Hubs- These nations are significant trade and investment hubs in Asia and globally. Their economies are deeply integrated into global supply chains, with substantial imports and exports influencing energy demands and environmental impacts. The influx of cross-border investment (FDI) into these countries further shapes their industrial landscapes and environmental policies, presenting unique challenges and opportunities for long-term environmental growth. Environmental Challenges and Policy Responses- Urbanization and Population Dynamics: Urbanization and population growth are significant drivers of energy consumption and environmental degradation in these countries. Rapid urbanization has increased energy demand for infrastructure development, transportation systems, and residential needs, contributing to carbon emissions and environmental pressures. Policy Innovation- Each country has implemented distinctive environmental policies and initiatives to address sustainability challenges. From sustainable energy incentives to stringent emissions regulations and green technology investments, their policy responses vary based on economic priorities, technological capabilities, and environmental imperatives. Comparative Analysis and Regional Dynamics- Regional Context: Analyzing these six countries allows for comparative analysis within the context of developed Asian economies. Their geographical proximity and shared economic interests create a conducive environment for examining common environmental challenges and evaluating the effectiveness of policy interventions. Global Influence- These nations greatly influence global environmental governance and climate negotiations. Their policy decisions and technological innovations have implications beyond regional boundaries, shaping international discourse on actions to mitigate climate change, long-term environmental growth goals, and global environmental stewardship. In conclusion, the selection of Japan, South Korea, Singapore, Hong Kong, China, and Israel as focal points of this study is justified by their economic significance, technological advancement, environmental challenges, policy responses, and regional dynamics within developed Asian contexts. By examining these countries, the study seeks to provide valuable insights into the complex interactions between economic activities, energy consumption patterns, and ecological sustainability, being supported by empirical evidence of policymaking and contributing to global efforts towards a sustainable future [1,10,12,14,15].

Understanding the complex interactions between economic activities, energy consumption, and ecological sustainability is paramount in contemporary discourse on global development. This study focuses on six developed Asian countries—Japan, South Korea, Singapore, Hong Kong, China, and Israel—from 2000 to 2020. The introduction provides a comprehensive discussion of vital environmental theories that underpin the study's framework, emphasizing their relevance and applicability in analyzing the environmental dynamics within these nations. Environmental Theories- Environmental Kuznets Curve (EKC): The Environmental Kuznets Curve proposes a hypothesized relationship between economic development and environmental quality. Initially articulated by Ref. [16], it suggests that environmental degradation worsens in the early stages of industrialization and economic development. However, environmental quality improves beyond a certain income level as societies prioritize ecological preservation, adopt cleaner technologies, and enforce stringent rules protecting the environment [17]. This theory is pivotal in understanding how economic development can eventually lead to ecological sustainability through policy interventions and technological advancements [9].

Energy Transition Theory- Energy transition theory focuses on shifting from fossil fuel dependence to sustainable energy sources to mitigate environmental impacts, particularly carbon emissions. It emphasizes the role of technological innovation, policy interventions, and market mechanisms in accelerating the adoption of sustainable energy solutions [18]. This theory is critical in examining the pathways for reducing greenhouse gas emissions and enhancing energy efficiency across developed economies like Asia. Ecological Modernization Theory- Ecological modernization theory posits that economic development and ecological preservation are not mutually exclusive but can be synergistic. It argues that technological advancements and environmental policies can drive the industrial transformation towards more sustainable practices, thereby decoupling economic development from environmental degradation [19]. This theory underscores the potential for innovation and policy innovation to foster long-term environmental growth in developed countries.

Application to Developed Asian Countries- Economic Significance and Policy Responses: The selected Asian countries—Japan, South Korea, Singapore, Hong Kong, China, and Israel—represent diverse economic landscapes characterized by rapid industrialization, technological innovation, and significant environmental challenges. Their experiences provide valuable case studies for testing these environmental theories in real-world contexts: Urbanization and Industrialization: Rapid urbanization and industrial growth have driven energy demand and environmental pressures in these nations, highlighting the relevance of the Environmental Kuznets Curve. Renewable Energy Adoption: Policies promoting sustainable energy adoption and efficiency align with the Energy Transition Theory, demonstrating pathways toward reducing carbon emissions while sustaining economic development [7].

Policy Innovations: Examples of stringent rules protecting the environment and green technology investments exemplify Ecological Modernization Theory, illustrating how policy interventions can foster ecological sustainability alongside economic development. Conclusion- By grounding the study in these foundational environmental theories, this research seeks to provide nuanced insights into the dynamics between economic activities, energy consumption patterns, and ecological sustainability in developed Asian countries. The comprehensive theoretical framework informs the empirical analysis of data from 2000 to 2020, offering a structured approach to understanding the complexities of environmental challenges and opportunities in the context of global long-term environmental growth goals [6].

The paper comprises five sections: part 1 is the introduction, part 2 presents the literature review, part 3 explains the data and research methodology, part 4 presents the research results, part 5 contains the discussion and part 6 is the conclusion.

## 2 Literature review

Global atmospheric warming is a pressing issue primarily attributable to environmental contaminants. The scientific community and governmental authorities are rigorously working to mitigate these pollution levels, aligning their efforts with the Sustainable Development Goals (SDGs). The main goals of the research are as follows: The objective is to identify and understand the various sources of CO2 emission. This issue includes natural sources like respiration, ocean release, volcanic emissions, and human-made sources like fossil fuel combustion, deforestation, and industrial processes. The objective of this investigation is to quantify the influence of individual determinants on overall CO2 output. The findings of this research could elucidate the significant contributors to the greenhouse effect and global temperature elevation. The other goals track changes and trends in carbon dioxide output over time. This goal can provide valuable insights into how human activities and natural processes affect CO2 levels [2,[20], [21], [22]]. Developing Predictive Models-to create models that predict future carbon dioxide output based on various determinants. This objective can be crucial for climate modelling and forecasting. They influence policymaking by providing data and insights. This goal could involve suggesting ways to reduce emissions from the most significant sources or advocating changes in human behaviour and industrial practices. The study investigates and analyzes potential strategies for reducing carbon dioxide output, including technological solutions, energy production and consumption changes, and carbon sequestration methods. To evaluate the success of various interventions to reduce carbon dioxide output [[23], [24], [25], [26]]. This problem can help refine these strategies and make them more effective. Promoting Sustainable Development-to contribute to developing sustainable technologies that can help reduce the effect of CO2 output on the environment. By achieving these objectives, the study can contribute to a more sustainable and resilient future, preserving the planet for generations [4,[27], [28], [29]].

Aghasafari et al. investigated the relationship between carbon dioxide output, exports, and cross-border investment (FDI) in the Middle East and North Africa (MENA) region [21]. Similarly, Al Afif et al. conducted a study on optimal sizing analysis for hybrid sustainable energy systems, using Al-Karak, Jordan, as a case study [22]. However, a gap exists in the current literature, as no research has yet utilized the Cobb-Douglas model to analyze the factors influencing ecological contamination in developed Asian countries. Additionally, there needs to be more examination of how sustainable energy consumption, FDI inflows, economic development, imports, exports, and population growth impact carbon dioxide output [3]. Consequently, this study explores the nexus between power usage, energy from fossil sources consumption, sustainable energy usage, FDI inflows, imports and exports of goods and services, economic development, population size, and carbon dioxide output across six developed Asian countries [24].

Imports refer to the transactions of goods purchased or sold between countries [26], while population size reflects the number of individuals residing in a specific geographical area at a given time. Population growth has wide-ranging effects on a nation's economy, social systems, and environmental conditions [25,26]. Carbon dioxide emissions refer to the release of CO2 into the atmosphere, primarily caused by human activities such as electricity generation and burning fossil fuels. CO2 is a significant greenhouse gas that exacerbates climate change and global warming [26]. The literature reveals various connections between these concepts. Increased power usage tends to promote economic development. However, if this electricity is predominantly derived from fossil fuels, it can produce higher carbon dioxide output, adversely affecting the environment and public health. Fossil fuel consumption is closely associated with elevated carbon dioxide output, contributing to global warming and rising sea levels [30]. The current study provides a novel contribution by demonstrating that sustainable energy consumption and economic development negatively impact carbon dioxide output, offering new insights into the dynamics of long-term environmental growth.

Research Gap- Previous studies have explored various dimensions of the relationship between economic development, energy consumption, and environmental outcomes. However, significant gaps persist. Timeliness and Contextual Relevance: Given the ongoing global climate crisis and the increasing urgency to address environmental challenges, updated empirical studies are critically needed to focus specifically on developed Asian economies [6,7]. These countries are pivotal players in global economic networks and face unique environmental management challenges. Integrated Analysis: While individual studies have examined aspects such as energy consumption or economic development, there is a lack of integrated analyses that consider the collective impact of multiple factors—such as FDI, trade dynamics, and population growth—on ecological contamination within these specific regional contexts [1,[31], [32], [33]].

Policy Implications: Existing literature often provides theoretical frameworks but fails to offer actionable insights for policymakers. This study seeks to bridge this gap by providing empirical evidence that can inform effective policy interventions to achieve economic prosperity and ecological sustainability. Contribution and Novelty- This study differs from previous research in several key aspects- Comprehensive Scope: By analyzing a comprehensive set of variables over a two-decade period, the study offers a holistic view of the interactions between economic activities, energy consumption patterns, and environmental outcomes in developed Asian countries. Methodological Rigor: The study utilizes advanced panel data regression techniques, and the research ensures robustness in the analysis of complex relationships and controls for potential confounding factors such as country-specific effects and time trends. Policy Relevance: The findings provide practical implications for policymakers by identifying effective strategies for transitioning towards cleaner energy sources, enhancing energy efficiency, and sustaining economic development [1,34,35].

Alignment with Recent Developments: This study is timely and relevant in light of current global trends. Technological Advancements: Innovations in sustainable energy and energy efficiency technologies create new opportunities to reduce environmental impacts while fostering economic development. Climate Commitments: International agreements like the Paris Agreement have increased the pressure on countries to adopt long-term environmental growth practices and lower their greenhouse gas emissions. Policy Innovations: Some nations have introduced pioneering policies to encourage sustainable energy adoption and ecological sustainability, offering valuable case studies for comparison. In conclusion, this study seeks to fill significant gaps in the literature by providing timely insights into the complex relationships between economic activities, energy consumption, and ecological sustainability in developed Asian countries. The findings will be supported by empirical evidence in policymaking and support global efforts to achieve long-term environmental growth goals [1,36,37].

In the literature review on the elements influencing carbon dioxide output, we must examine various themes, such as energy consumption, industrialization, population growth, economic development, urbanization, transportation, land-use changes, and technological advancements. Research shows a significant correlation between energy consumption and carbon dioxide output [30,[38], [39], [40]]. Carbon dioxide output increases as nations consume more energy, especially fossil fuels [27,28,41]. Studies also suggest that population growth can increase carbon dioxide output as more people require energy, goods, and services, increasing fossil fuel consumption [29,42,43]. Urbanization often leads to increased energy use and, thus, higher carbon dioxide output, especially in rapidly urbanizing regions like Asia and Africa. However, urban areas can also benefit from economies of scale in infrastructure like public transport and energy-efficient buildings [[44], [45], [46], [47]].

Industrial processes contribute significantly to global carbon dioxide output. These processes often involve burning fossil fuels for energy, directly emitting CO2 into the atmosphere. Technological improvements can help reduce carbon dioxide output by improving energy efficiency and enabling the development and deployment of sustainable energy sources [48,49]. The transportation industry is a significant contributor to carbon dioxide output, mainly through the burning of gasoline and diesel fuels in vehicles. Deforestation and land-use changes contribute to carbon dioxide output by reducing the Earth's capacity to absorb CO2 and releasing stored carbon when trees are cut down. Economic development typically raises carbon dioxide output due to increased industrial activities and energy consumption. However, the Environmental Kuznets Curve (EKC) hypothesis suggests that wealthier countries can afford to invest in cleaner technologies beyond a certain income level, leading to a decline in carbon dioxide output [[50], [51], [52], [53]].

This brief literature review provides the general vital themes related to carbon dioxide output. However, it is essential to note that these factors do not operate in isolation. They are often interrelated, and their impacts vary significantly depending on each country or region's specific context and circumstances. They have studied the green industry in Vietnam [49]. He focused on the importance of decreasing ecological contamination by using the standards of environment management of SMEs' green sectors, which can help to reduce carbon dioxide output. Fernandes et al. researched urban metabolism-based approaches for promoting the circular economy in building refurbishment [51]. They noted that the circular economy can help to reduce ecological contamination as it encourages the use of sustainable energy. The escalating carbon dioxide (CO2) emissions and their subsequent impact on worldwide shifts in climate patterns have been a focal point for scientific and policy-oriented discussions. This literature review examines the determinants influencing carbon dioxide output, as presented in the existing body of scholarly research. The review organizes these determinants into several categories, including economic, technological, industrial, and sociopolitical factors [40]. Gross Domestic Product (GDP)- numerous studies have identified a positive nexus of a country's GDP and carbon dioxide output. The underlying principle is that economic activities often entail energy consumption, predominantly from fossil fuels, leading to emissions [28]. Foreign Direct Investment (FDI)- FDI can have a dual impact. While it can boost the economy and modernize industries, it might also lead to increased emissions if the investment is in fossil fuel-intensive [47].

Technological Factors- Energy Efficiency: Advancements in energy efficiency technology can significantly reduce emissions. However, the so-called "rebound effect" might counter these gains, as better efficiency often leads to increased consumption [48]. Renewable energy-adopting sustainable energy sources like wind, solar, and hydroelectric power has shown promise in reducing carbon dioxide output [26]. Industrial Factors-the type of industry-different industries have varying carbon dioxide output levels. For example, manufacturing and transportation are often high-emitting sectors [30]. Industrial Processes: Certain industrial processes, like cement production, are inherently carbon-intensive and significantly contribute to emissions [25]. Sociopolitical Factors- Public Awareness and Education: Public awareness and education levels have been linked to emissions, as more educated societies support environmentally-friendly policies [54]. Government Policies and Regulations-effective governmental policies, including carbon taxes and cap-and-trade systems, have effectively reduced carbon dioxide output [55]. Understanding the multiplicity of elements influencing carbon dioxide output is crucial for academic and practical purposes. As the scientific community moves toward a more integrated understanding of these determinants, this literature review is a foundational reference for researchers and policymakers.

Firth et al. investigated the carbon and CO2 flux in soybean cropping systems under cover crop and no-till management in a mid-South American state [53], concluding that production increased carbon dioxide output in this region of the USA. Flammini et al. found that green production can reduce carbon dioxide output [56]. Hu et al. studied the effects of cover crops and soil amendments in Mississippi corn systems, reporting increased carbon dioxide output due to these practices [57]. Huang et al. explored the link between sustainable energy and carbon dioxide output in major energy-consuming countries, finding that greater sustainable energy use reduces pollution. Johnathon et al. developed a hedge-based energy market model to manage sustainable energy intermittency, proposing solutions for improving market efficiency. Joo et al. studied the relationship between FDI, economic development, and host country characteristics in BRICS nations, concluding that FDI inflows and economic development contribute to ecological contamination. Khan et al. examined the global relationship between innovations, energy consumption, and carbon dioxide output, highlighting these links. Le et al. used a nonlinear ARDL co-integration approach to analyze FDI, pollution, and economic development, concluding that both FDI and pollution can foster growth. Liem et al. studied greenhouse gas emissions from organic fertilizer use in lime cultivation in Vietnam's Mekong Delta, finding that organic fertilizers can lower carbon dioxide output [[58], [59], [60]].

Madani et al. researched the correlation between air pollution, poverty, smoking, and disease in New York State, identifying a link between these factors [61,62]. Martí-Ballester et al. found that sustainable mutual funds can drive clean energy investments, boost green economy growth, and reduce carbon dioxide output. Nguyen examined Vietnam's export-driven carbon emissions, noting that rising exports decreased carbon dioxide output [[63], [64], [65]]. Another study by Nguyen Thi Ngoc explored how FDI inflows contribute to ecological contamination in Vietnam. Nguyen et al. focused on financial development and sustainable energy in Southeast Asia, emphasizing the role of organic waste materials in reducing pollution. Overland et al. analyzed whether sustainable energy is more evenly distributed than fossil fuels and concluded that it enhances long-term environmental growth [40,[66], [67], [68], [69], [70], [71]].

Raihan et al. examined the links between Russia's energy use, industrialization, forest area, and carbon dioxide output, concluding that green energy and forests reduce carbon dioxide output [72]. They also analyzed the nexus of Peru's economic development, sustainable energy, agriculture, and carbon dioxide output, noting that growth increases emissions. Ram et al. explored Delhi's shift toward 100 % sustainable energy and referenced the SDGs as essential to achieving this goal. Shahzad et al. found that exports can increase carbon dioxide output in developed and developing countries. Shang et al. focused on how climate policies and uncertainty affect energy demand in the US, recommending an emphasis on sustainable energy. Stjepanovic et al. assessed a new green GDP database, concluding that sustainable energy boosts the green economy. Sun et al. studied long-term environmental growth in China, finding that increased sustainable energy use improves ecological preservation [[73], [74], [75], [76], [77], [78], [79], [80], [81], [82], [83], [84], [85]]. These goals are integral to the 2030 Agenda for Sustainable Development. These interrelated objectives tackle global challenges across economic, social, and environmental dimensions. Achieving these goals requires action and collaboration from governments, businesses, civil society, and individuals worldwide.

Carbon dioxide output has become a pressing environmental concern due to its contribution to climate change. The increase in atmospheric CO2 levels, primarily from human activities, drives global warming and the associated environmental impacts. This literature review explores the present understanding of CO2 output, its sources, and the implications for climate change [86]. Numerous studies have highlighted the primary sources and drivers of CO2 output. Fossil fuel energy combustion is the most significant contributor, accounting for approximately 75 % of global emissions. This goal includes electricity generation, transportation, industrial processes, and residential energy use emissions. Deforestation and land-use changes also significantly contribute to around 20 % of carbon dioxide output. The remaining emissions stem from various sectors, such as agriculture, cement production, and waste management [40]. The persistent carbon dioxide (CO2) emission into the atmosphere substantially influences climatic alterations. CO2 serves as a greenhouse gas, confining thermal energy within the Earth's atmospheric envelope, thereby contributing to elevations in global temperature. The Intergovernmental Panel (IP) on Climate Change (CC) has emphasized the correlation between escalating concentrations of CO2 and observable modifications in temperature, precipitation regimes, sea-level fluctuations, and incidents of extreme meteorological phenomena. These impacts have far-reaching consequences for ecosystems, human health, agriculture, and global economies [40,87].

Multiple ameliorative approaches have been examined to confront the complexities associated with carbon dioxide (CO2) emissions. Alternative energy modalities, including photovoltaic, hydroelectric, and wind-based technologies, present a viable substitute for fossil fuel utilization, thereby diminishing carbon dioxide output during electrical power production. Energy efficiency, such as building insulation and fuel-efficient transportation, is vital to reducing emissions. Additionally, afforestation and reforestation programs can help sequester carbon dioxide by promoting the uptake of CO2 through photosynthesis [88]. Technological advancements in reality, such as carbon capture and storage (CCS), have gained attention as potential solutions for reducing carbon dioxide output from fossil fuel-based industries. CCS involves capturing carbon dioxide output at its source, transporting it, and securely storing it underground or in other long-term repositories. While CCS shows promise, its large-scale implementation and associated costs remain challenges [89]. The literature emphasizes the importance of effective policy frameworks and international cooperation in addressing CO2 output. The Paris Agreement, adopted in 2015, outlines a global effort, and therefore, it limits world warming to below 2 °C and above pre-industrial levels. Nationally Determined Contributions (NDCs) are critical in guiding countries' emission reduction targets and actions. The literature suggests that solid policies, such as carbon pricing mechanisms and regulations promoting clean technologies, are essential for achieving emission reduction goals [40,90].

The literature reviewed underscores the significant role of CO2 output in driving climate change. Fossil fuel combustion, deforestation, and land-use changes are the primary sources of carbon dioxide output. The impacts of these emissions are far-reaching, affecting ecosystems, human well-being, and socio-economic systems. Mitigation strategies have been proposed to reduce carbon dioxide (CO2) emissions, including adopting sustainable energy, efficiency, and capture technologies. International cooperation and robust policy frameworks are essential for achieving emission reduction targets and mitigating the effects of CO2 output (CO2) on climate change [26,30].

In summary, the recent research does not focus on using the Cobb-Douglas model to estimate the elements influencing ecological contamination in developed Asian countries. Previous research shows no negative relationship between FDI, green energy, and ecological contamination in developed Asian countries. Therefore, this paper focuses on using the Cobb-Douglas model to analyze the elements influencing CO2 Emissions and, thus, the achievement of some SDGs in developed Asian countries. The data and research methodology are presented in the following section.

## 3 Data and methodology

### 3.1 Data

Data Selection and Justification- This study uses panel data from 2000 to 2020 for six developed Asian countries: Japan, South Korea, Singapore, Hong Kong, China, and Israel. The section explains the data sources, variables, and the rationale for selecting this time frame. Data Sources: The data come from well-established international databases to ensure reliability and consistency:

CO2 Emissions: Sourced from the World Bank's World Development Indicators (WDI).

Electricity Consumption: Obtained from the International Energy Agency (IEA).

Fossil Fuel Use: From the BP Statistical Review of World Energy.

Renewable Energy Adoption: Collected from the International Renewable Energy Agency (IRENA).

Foreign Direct Investment (FDI): Sourced from the United Nations Conference on Trade and Development (UNCTAD).

Imports and Exports: Data from the World Trade Organization (WTO).

Economic Growth (GDP growth): The International Monetary Fund (IMF) provides.

Population: Collected from the United Nations Department of Economic and Social Affairs (UNDESA) [1,9,10,12].

Variables- The following crucial factors are used in the analysis:

CO2 Emissions (CO2): Metric tons of carbon dioxide output per capita. Electricity Consumption (ELEC): Kilowatt-hours (kWh) of electricity consumed per capita. Fossil Fuel Use (FFUEL): Percentage of total energy consumption derived from fossil fuels. Renewable Energy Adoption (RENE): Percentage of total energy consumption derived from renewable sources. Foreign Direct Investment (FDI): Net inflows of FDI as a percentage of GDP. Imports (IM): Value of imports as a percentage of GDP. Exports (EX): Value of exports as a percentage of GDP. Economic Growth (GDP growth): Annual percentage growth rate of GDP. Population (POP): Total population in millions.

The rationale for the Time Frame (2000–2020)- While extending the data range from 1990 to 2020 would offer a more extended trend analysis, several reasons justify the focus on the period from 2000 to 2020: Data Consistency and Availability: The availability and consistency of reliable data for some variables significantly improved after 2000. Many countries and international organizations started systematic and comprehensive data collection in the early 2000s, ensuring higher quality and comparability. Technological Advancements: The early 2000s marked significant technological advancements, particularly in the energy sector. The adoption of sustainable energy technologies and improvements in energy efficiency gained momentum during this period, providing a relevant context for analyzing their impact on ecological contamination. Globalization and Economic Changes: The 2000s witnessed substantial changes in global trade patterns, FDI flows, and economic integration. These changes are crucial for understanding the dynamics between economic activities and ecological contamination in the context of developed Asian countries. Environmental Policies: Many countries implemented vital environmental policies and international agreements, such as the Kyoto Protocol and the Paris Agreement, starting in the late 1990s and early 2000s. Analyzing data from 2000 onwards allows for an assessment of the impact of these policies on environmental outcomes [2,4,6,7].

Data Preparation and Cleaning- Data from the identified sources were extracted, cleaned, and processed to ensure consistency and accuracy. Missing values were addressed using interpolation or imputation techniques where feasible, and variables were standardized to enable comparability across countries. Summary- By focusing on the period from 2000 to 2020, this study leverages a time frame characterized by significant technological, economic, and policy developments. This approach provides a robust basis for analyzing the complex relationships between power usage, fossil fuel use, sustainable energy adoption, FDI, imports, exports, economic development, population, and ecological contamination in six developed Asian countries. The chosen time frame ensures data quality and relevance, facilitating a comprehensive and accurate assessment of the studied relationships.

This paper also uses data from the World Bank for the period 20002020 as follows:

Carbon dioxide output is expressed in millions of tons per year, indicating ecological contamination. The study assumes that higher carbon dioxide output signifies more significant environmental degradation. Electricity consumption refers to each country's total annual energy use, encompassing all energy sources like coal, water, wind, fossil fuels, and solar power, measured in terawatt-hours (TWh). Fossil fuel consumption reflects the total non-sustainable energy usage over a year, primarily from burning fossil fuels. Renewable energy consumption measures the total green energy used annually in each country, including water, wind, and solar energy. Foreign direct investment (FDI) inflows are recorded as the total amount of FDI entering each country by year-end, measured in US dollars. Exports of goods and services represent the total value of exports by the end of the year, also measured in US dollars. At the same time, imports reflect the total value of imported goods and services, measured in US dollars. GDP measures economic development in US dollars, and population is recorded as the total number of people living in each country at the end of the year.

Data Analysis- The data analysis for this study involved several stages, including data preparation, exploratory data analysis, correlation analysis, panel data regression, and diagnostic tests. This section describes each step and the statistical tests conducted before arriving at the final model. Data Preparation- Data Cleaning and Transformation: Data Cleaning- The raw data were cleaned to handle missing values, outliers, and inconsistencies. Missing values were addressed through interpolation or imputation techniques where feasible. Outliers were detected and examined for possible data entry errors or anomalies. Standardization: Variables were standardized to enable comparability across countries and periods. This issue involved normalizing data to a standard scale, particularly for variables measured in different units [1]

Exploratory Data Analysis (EDA)- Descriptive Statistics: Descriptive statistics, including mean, standard deviation, minimum, and maximum values, were computed for each variable. This issue provided an initial understanding of the data distribution and central tendencies. Visualization- Histograms and Box Plots: These visualizations were used to examine the distribution of each variable and identify potential outliers. Line Charts: Time series line charts were plotted for each country to observe trends from 2000 to 2020. Scatter Plots: Scatter plots were created to visualize relationships between pairs of variables, helping to identify potential correlations and nonlinear patterns. Correlation Analysis- A correlation matrix was computed to examine the linear relationships between the variables. Pearson correlation coefficients were calculated to identify the strength and direction of the relationships. This step helped to identify multicollinearity issues and provided a preliminary understanding of how the variables are related. Panel Data Regression Analysis- Model Selection: Given the panel nature of the data (multiple countries over multiple years), panel data regression techniques were employed. Two primary models were considered: Fixed-Effects Model (FEM): Controls for unobserved heterogeneity by allowing the intercept to vary across countries. This model is useful when individual country effects are correlated with the independent variables [12].

The Random-Effects Model (REM) assumes that the individual-specific effects are not correlated with the independent variables, making it suitable when variations across countries are random and unrelated to the predictors. Model Estimation: Both fixed-effects and random-effects models were applied in this study. The independent variables included power usage (ELEC), fossil fuel use (FFUEL), sustainable energy adoption (RENE), cross-border investment (FDI), imports (IM), exports (EX), economic development (GDP), and population (POP). Diagnostic Tests: Hausman Test: This test was performed to decide between the fixed-effects and random-effects models. The null hypothesis suggests that the random-effects model is appropriate. A significant test statistic indicates that the fixed-effects model is preferred. Breusch-Pagan LM Test: Used to test for heteroskedasticity. The null hypothesis states no heteroskedasticity, meaning the error variance remains constant. Wooldridge Test for Autocorrelation: This test was conducted to check for autocorrelation in panel data. The null hypothesis assumes no first-order autocorrelation exists in the residuals [9,10].

Multicollinearity Check- Variance Inflation Factors (VIF) were calculated to check for multicollinearity among the independent variables. A VIF value exceeding 10 indicates a high level of multicollinearity, which could distort the regression results. Robustness Checks- Sensitivity Analysis: the research ensures the robustness of the results; sensitivity analyses were conducted by varying the model specifications and including/excluding certain variables. These checks helped confirm the stability and reliability of the findings. Robust Standard Errors- Robust standard errors were estimated to account for potential heteroskedasticity and autocorrelation, providing more reliable inference. Summary of Results- Based on the diagnostic tests and robustness checks, the final model selection was the fixed-effects model. This model provided the most consistent and reliable estimates, capturing the country-specific effects and controlling for unobserved heterogeneity. By following this comprehensive data analysis approach, the study was able to uncover significant interdependencies among the variables and provide valuable insights into the nexus between power usage, fossil fuel use, sustainable energy adoption, FDI, imports, exports, economic development, population, and ecological contamination in six developed Asian countries [2,4,6,7].

### 3.2 Methodology

**Fig. 1:**
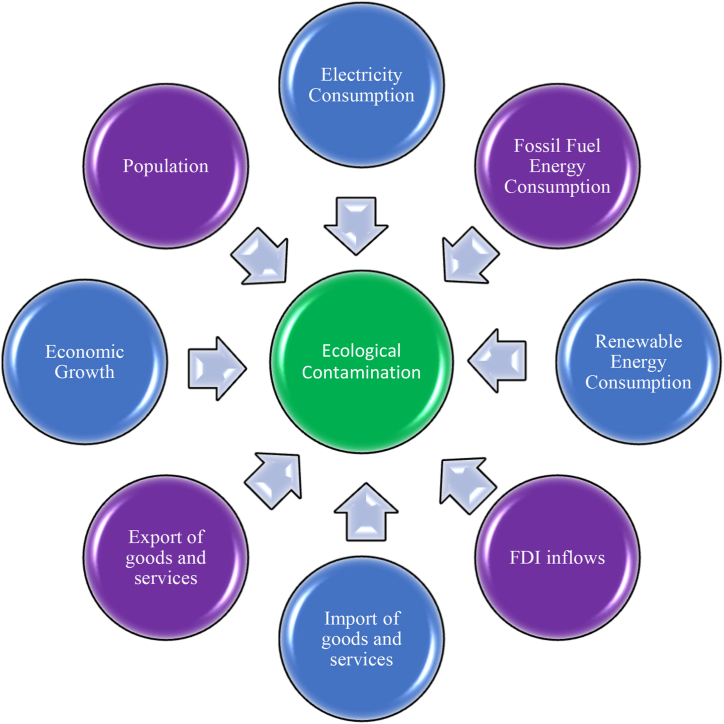
The research graphical abstract. (Source: compiled by authors).

The research graphical abstract is illustrated in Fig. 1:

The research uses the function given in Equation (1):

(1) CO2 = f(a1, a2, a3, a4, a5, a6, a7, a8, a9 …)

Where CO2 is carbon dioxide output, the dependent variable. It is a measure of ecological contamination; a1 is power usage, an independent variable, measured in TWh; a2 is energy from fossil sources consumption, an independent variable, measured in TWh; a3 is sustainable energy consumption, an independent variable, measured in TWh, and as a percentage of total power usage, a4 is FDI inflows, an independent variable, measured in USD dollars, a5 is Exports of goods and services, an independent variable, measured in USD dollars, a6 is Imports of goods and services, an independent variable, measured in US dollars, a7 is GDP, an independent variable; measured in USD dollars, a8 is population, an independent variable, measured as the number of persons.

The paper uses the Cobb-Douglas model to estimate carbon dioxide output as in Equation (2):

(2) $$Q_{i,t}=A.{a1}_{i,t}^{\alpha 1}{a2}_{i,t}^{\alpha 2}{a3}_{i,t}^{\alpha 3}{a4}_{i,t}^{\alpha 4}{a5}_{i,t}^{\alpha 5}{a6}_{i,t}^{\alpha 6}{a7}_{i,t}^{\alpha 7}{a8}_{i,t}^{\alpha 8}+\varepsilon_{i,t}$$

The elasticity of carbon dioxide output to the independent variable a1 (power usage) is measured by equation (3) as follows:

(3) $$E_{a1}=\frac{dQ_{i,t}}{d{a1}_{i,t}}x\;\frac{{a1}_{i,t}}{Q_{i,t}}=A.\alpha 1.\;{a1}_{i,t}^{\alpha 1-1}{a2}_{i,t}^{\alpha 2}{a3}_{i,t}^{\alpha 3}{a4}_{i,t}^{\alpha 4}{a5}_{i,t}^{\alpha 5}{a6}_{i,t}^{\alpha 6}{a7}_{i,t}^{\alpha 7}{a8}_{i,t}^{\alpha 8}.\frac{a1}{A.{a1}_{i,t}^{\alpha 1}{a2}_{i,t}^{\alpha 2}{a3}_{i,t}^{\alpha 3}{a4}_{i,t}^{\alpha 4}{a5}_{i,t}^{\alpha 5}{a6}_{i,t}^{\alpha 6}{a7}_{i,t}^{\alpha 7}{a8}_{i,t}^{\alpha 8}}$$

The elasticity of carbon dioxide output to the independent variable a2 (energy from fossil sources consumption) is measured by equation (4) as follows:

(4) $$E_{a2}=\frac{dQ_{i,t}}{d{a2}_{i,t}}x\;\frac{{a2}_{i,t}}{Q_{i,t}}=A.\alpha 2.\;{a1}_{i,t}^{\alpha 1}{a2}_{i,t}^{\alpha 2-1}{a3}_{i,t}^{\alpha 3}{a4}_{i,t}^{\alpha 4}{a5}_{i,t}^{\alpha 5}{a6}_{i,t}^{\alpha 6}{a7}_{i,t}^{\alpha 7}{a8}_{i,t}^{\alpha 8}\frac{a2}{A.{a1}_{i,t}^{\alpha 1}{a2}_{i,t}^{\alpha 2}{a3}_{i,t}^{\alpha 3}{a4}_{i,t}^{\alpha 4}{a5}_{i,t}^{\alpha 5}{a6}_{i,t}^{\alpha 6}{a7}_{i,t}^{\alpha 7}{a8}_{i,t}^{\alpha 8}}=\alpha 2$$

The elasticity of carbon dioxide output to the independent variable a3 (sustainable energy consumption) is measured by equation (5) as follows:

(5) $$E_{a3}=\frac{dQ_{i,t}}{d{a3}_{i,t}}x\;\frac{{a3}_{i,t}}{Q_{i,t}}=A.\alpha 3.\;{a1}_{i,t}^{\alpha 1}{a2}_{i,t}^{\alpha 2}{a3}_{i,t}^{\alpha 3-1}{a4}_{i,t}^{\alpha 4}{a5}_{i,t}^{\alpha 5}{a6}_{i,t}^{\alpha 6}{a7}_{i,t}^{\alpha 7}{a8}_{i,t}^{\alpha 8}\frac{a3}{A.{a1}_{i,t}^{\alpha 1}{a2}_{i,t}^{\alpha 2}{a3}_{i,t}^{\alpha 3}{a4}_{i,t}^{\alpha 4}{a5}_{i,t}^{\alpha 5}{a6}_{i,t}^{\alpha 6}{a7}_{i,t}^{\alpha 7}{a8}_{i,t}^{\alpha 8}}=\alpha 3$$

The elasticity of carbon dioxide output to the independent variable a4 (FDI inflows) is measured by equation (6) as follows:

(6) $$E_{a4}=\frac{dQ_{i,t}}{d{a4}_{i,t}}x\;\frac{{a4}_{i,t}}{Q_{i,t}}=A.\alpha 4.\;{a1}_{i,t}^{\alpha 1}{a2}_{i,t}^{\alpha 2}{a3}_{i,t}^{\alpha 3}{a4}_{i,t}^{\alpha 4-1}{a5}_{i,t}^{\alpha 5}{a6}_{i,t}^{\alpha 6}{a7}_{i,t}^{\alpha 7}{a8}_{i,t}^{\alpha 8}.\frac{a4}{A.{a1}_{i,t}^{\alpha 1}{a2}_{i,t}^{\alpha 2}{a3}_{i,t}^{\alpha 3}{a4}_{i,t}^{\alpha 4}{a5}_{i,t}^{\alpha 5}{a6}_{i,t}^{\alpha 6}{a7}_{i,t}^{\alpha 7}{a8}_{i,t}^{\alpha 8}}=\alpha 4$$

The elasticity of carbon dioxide output to the independent variable a5 (exports of goods and services) is measured by equation (7) as follows:

(7) $$E_{a5}=\frac{dQ_{i,t}}{d{a5}_{i,t}}x\;\frac{{a5}_{i,t}}{Q_{i,t}}=A.\alpha 5.\;{a1}_{i,t}^{\alpha 1}{a2}_{i,t}^{\alpha 2}{a3}_{i,t}^{\alpha 3}{a4}_{i,t}^{\alpha 4}{a5}_{i,t}^{\alpha 5-1}{a6}_{i,t}^{\alpha 6}{a7}_{i,t}^{\alpha 7}{a8}_{i,t}^{\alpha 8}\frac{a5}{A.{a1}_{i,t}^{\alpha 1}{a2}_{i,t}^{\alpha 2}{a3}_{i,t}^{\alpha 3}{a4}_{i,t}^{\alpha 4}{a5}_{i,t}^{\alpha 5}{a6}_{i,t}^{\alpha 6}{a7}_{i,t}^{\alpha 7}{a8}_{i,t}^{\alpha 8}}=\alpha 5$$

The elasticity of carbon dioxide output to the independent variable a6 (imports of goods and services) is measured by equation (8) as follows:

(8) $$E_{a6}=\frac{dQ_{i,t}}{d{a6}_{i,t}}x\;\frac{{a6}_{i,t}}{Q_{i,t}}=A.\alpha 6.\;{a1}_{i,t}^{\alpha 1}{a2}_{i,t}^{\alpha 2}{a3}_{i,t}^{\alpha 3}{a4}_{i,t}^{\alpha 4}{a5}_{i,t}^{\alpha 5}{a6}_{i,t}^{\alpha 6-1}{a7}_{i,t}^{\alpha 7}{a8}_{i,t}^{\alpha 8}\frac{a6}{A.{a1}_{i,t}^{\alpha 1}{a2}_{i,t}^{\alpha 2}{a3}_{i,t}^{\alpha 3}{a4}_{i,t}^{\alpha 4}{a5}_{i,t}^{\alpha 5}{a6}_{i,t}^{\alpha 6}{a7}_{i,t}^{\alpha 7}{a8}_{i,t}^{\alpha 8}}=\alpha 6$$

The elasticity of carbon dioxide output to the independent variable a7 (economic development) is measured by equation (9) as follows:

(9) $$E_{a7}=\frac{dQ_{i,t}}{d{a1}_{i,t}}x\;\frac{{a7}_{i,t}}{Q_{i,t}}=A.\alpha 7.\;{a1}_{i,t}^{\alpha 1}{a2}_{i,t}^{\alpha 2}{a3}_{i,t}^{\alpha 3}{a4}_{i,t}^{\alpha 4}{a5}_{i,t}^{\alpha 5}{a6}_{i,t}^{\alpha 6}{a7}_{i,t}^{\alpha 7-1}{a8}_{i,t}^{\alpha 8}\frac{a7}{A.{a1}_{i,t}^{\alpha 1}{a2}_{i,t}^{\alpha 2}{a3}_{i,t}^{\alpha 3}{a4}_{i,t}^{\alpha 4}{a5}_{i,t}^{\alpha 5}{a6}_{i,t}^{\alpha 6}{a7}_{i,t}^{\alpha 7}{a8}_{i,t}^{\alpha 8}}=\alpha 7$$

The elasticity of carbon dioxide output to the independent variable a8 (population) is measured by equation (10) as follows:

(10) $$E_{a8}=\frac{dQ_{i,t}}{d{a8}_{i,t}}x\;\frac{{a8}_{i,t}}{Q_{i,t}}=A.\alpha 8.\;{a1}_{i,t}^{\alpha 1}{a2}_{i,t}^{\alpha 2}{a3}_{i,t}^{\alpha 3}{a4}_{i,t}^{\alpha 4}{a5}_{i,t}^{\alpha 5}{a6}_{i,t}^{\alpha 6}{a7}_{i,t}^{\alpha 7}{a8}_{i,t}^{\alpha 8-1}\frac{a8}{A.{\beta 1}_{i,t}^{\alpha 1}{\beta 2}_{i,t}^{\alpha 2}{\beta 3}_{i,t}^{\alpha 3}{\beta 4}_{i,t}^{\alpha 4}{\beta 5}_{i,t}^{\alpha 5}{\beta 6}_{i,t}^{\alpha 6}{\beta 7}_{i,t}^{\alpha 7}{\beta 8}_{i,t}^{\alpha 8}}=\alpha 8$$

In Equation (2), the research logarithm on both sides of the Equation has Equation (11) as follows:

(11) Ln(Q_i,t_) = A + α_1_ Ln(a_1i,t_) + α_2_Ln(a_2i,t_) + α_3_ Ln(a_3i,t_) + α_4_ Ln(a_4i,t_) + α_5_ Ln(a_5i,t_) + α_6_ Ln(a_6i,t_) + α_7_ Ln(a_7i,t_) + α_8_ Ln(a_8i,t_) + e_i,t_

This paper assumes that the above Equation illustrates the relationship between the dependent variable, carbon dioxide output, and the independent variables. It could be described as follows:

Q is carbon dioxide output, the dependent variable; it represents ecological contamination, and a is the constant. It means that carbon dioxide output is when the independent variables (a1, a2, a3, a4, a5, a6, a7, and a8) equal zero. The research hypotheses are as follows.

H1 Electricity consumption (ECO) positively affects ecological contamination.

H2 Fossil fuel energy consumption (FFU) positively affects ecological contamination.

H3 Renewable energy consumption (REC) negatively affects ecological contamination.

H4 FDI inflows negatively affect ecological contamination.

H5 The exports positively affect ecological contamination.

H6 The imports positively affect ecological contamination.

H7 Economic Growth negatively affects ecological contamination.

H8 The population positively affects ecological contamination.

The independent variables used in the model are presented in Table 1 as follows.

**Table 1:** The independent parameters used in the Cobb-Douglas model.

Variable	Concept	Relationship
a_1_	Electricity consumption	+
a_2_	Fossil fuel	+
a_3_	Green energy	–
a_4_	Foreign direct investment	–
a_5_	The exports	+
a_6_	The imports	+
a_7_	Economic Growth	–
a_8_	Population	+

#### 3.2.1 Research and data methodology

Model Building Process: Variable Selection: The study employs a panel data regression framework to explore the relationship between various factors and carbon dioxide output in six developed Asian nations from 2000 to 2020. Dependent variable: carbon dioxide output (CO2) per capita, representing ecological contamination. Independent Variables: These include power usage (ELEC) per capita, fossil fuel use percentage (FFUEL), sustainable energy consumption percentage (RENE), FDI inflows as a share of GDP, imports (IM) and exports (EX) as percentages of GDP, GDP growth rate (GDP), and total population (POP) in millions [1,35,91].

Model Specification: Fixed-Effects Model (FEM): Controls for constant country-specific effects that may impact carbon dioxide output, particularly when these effects correlate with the independent variables. Random-Effects Model (REM): Assumes that country-specific effects are random and uncorrelated with the independent variables, making it appropriate when country-specific factors vary randomly. Empirical Approach: Data Preparation: The study involves cleaning and organizing data from reliable international sources for consistency. Descriptive Analysis: Descriptive statistics and visual representations are used to understand variable distributions over time and across countries. Correlation Analysis: Pearson correlation is applied to examine linear relationships between variables and check for multicollinearity. Model Estimation: Both FEM and REM are applied to evaluate the impact of the independent variables on carbon dioxide output [1,36,37,92].

Model Testing Approach: Using panel data regression models, this study aligns with previous research investigating the links between economic activities, energy consumption, and ecological contamination. Studies of similar nature often use FEM and REM to account for unobserved heterogeneity and verify results. Comparison with Previous Studies: The methodology is consistent with studies examining how energy consumption patterns, economic activities, and policy measures influence ecological contamination. FEM and REM are widely used in similar contexts to address unobserved factors and ensure result robustness across different models. Conclusion: By employing rigorous panel data regression techniques and comprehensive data sources, this study seeks to provide empirical insights into the relationship between economic activities, energy consumption, and ecological contamination in developed Asian countries. The approach builds on established methods while addressing critical gaps in understanding the factors driving carbon dioxide output and offering guidance for long-term environmental growth policies [1,93,94].

Theoretical Underpinnings: Environmental Kuznets Curve (EKC): Proposes an inverted U-shaped relationship between economic development and environmental degradation. Initially, economic development worsens environmental conditions, but environmental quality improves beyond a certain income level as cleaner technologies and stricter regulations are adopted [17,95,96]. Energy Transition Theory: Highlights the shift from fossil fuels to sustainable energy as essential for reducing environmental impacts, emphasizing policies that encourage sustainable energy adoption and efficiency. Variable Justification: Electricity Consumption (ELEC): Reflects energy demand and economic activity, with higher consumption often linked to industrialization and increased carbon dioxide output. Fossil Fuel Use (FFUEL) and Renewable Energy Adoption (RENE): Fossil fuel reliance leads to higher carbon dioxide output, while sustainable energy adoption can lower environmental impacts by replacing carbon-intensive energy sources. Foreign Direct Investment (FDI): FDI can affect environmental quality through technology transfers and industrial growth, but without strict environmental protection, it may increase pollution. Trade Dynamics (IM and EX): Imports and exports can impact environmental outcomes by influencing production and resource use. Increased trade can lead to higher emissions due to transportation and industrial activities [16,97].

Economic Growth (GDP) and Population (POP)- Economic Growth and population dynamics drive energy demand and consumption patterns. Rapid economic development often exacerbates environmental pressures, while population growth influences resource utilization and waste generation [28,98]. Contribution to Literature- This study builds on existing literature by offering several novel contributions: Integrated Analysis: Integrates multiple variables within a comprehensive analytical model to provide a holistic understanding of ecological contamination drivers in developed Asian countries. Empirical Rigor: Applies advanced panel data regression techniques to quantify the relationships between economic activities, energy consumption, and carbon dioxide output, enhancing methodological robustness and reliability. Policy Relevance: Provides insights supported by empirical evidence for policymakers to formulate effective strategies for long-term environmental growth and environmental management in the context of developed Asian economies. This study advances scientific understanding by anchoring variable selection in established theories and addressing gaps in prior research through rigorous empirical analysis. It offers practical implications for achieving ecological sustainability in the face of economic development and globalization.

## 4 Results

The nexus between FDI and economic development has recently been extensively researched. However, there needs to be more research that examines the nexus between power usage, energy from fossil sources consumption, sustainable energy consumption, FDI inflows, exports and imports of goods and services, economic development, population size, and ecological contamination. Therefore, the manuscript seeks to fill this gap and focus on these relationships in six developed Asian countries. The study analyses the elements influencing the environment in these selected countries.

The statistics are illustrated in Table 2:

**Table 2:** The descriptive statistics of the variables in the Cobb-Douglas regression model.

Variable	Obs	Mean	Std. dev.	Min	Max
Ln CO_2_ emissions	126	5.681412	1.945432	3.410223	9.274993
Ln electricity consumption	126	8.785816	0.4818639	6.97764	9.347778
Ln fossil fuel energy consumption	126	10.6936	0.6558968	9.058302	12.06804
Renewable energy consumption	126	2.87444	3.651905	0	14.24287
Ln FDI inflows	124	28.59407	1.300247	24.72506	31.00151
Ln Exports	126	26.77828	0.958415	24.66308	28.89756
Ln Imports	126	26.68063	0.9711608	24.49285	28.57263
Ln GDP	126	27.43318	1.48904	25.22079	30.32041
Ln Population	126	17.40334	2.001547	15.20875	21.06763

Table 2 presents data spanning from 2000 to 2020, encompassing 126 observations. The dependent variable Q represents carbon dioxide output, expressed in natural logarithm (Ln) terms. The average Ln carbon dioxide output stands at 5.68 million tons, with a peak of 9.27 million tons in 2020 and a low of 3.41 million tons in 2000. The first independent variable, a1, signifies power usage, expressed in Ln terms. The mean Ln power usage is 8.785 TWh, reaching a maximum of 9.35 TWh in 2020 and a minimum of 6.97 TWh in 2000. The second independent variable *a*2 pertains to energy from fossil sources consumption, with a mean Ln value of 10.69 million tons, peaking at 12.07 TWh in 2020 and dropping to 9.06 TWh in 2000.

The third independent variable, *a*3, measures sustainable energy consumption, which averages 2.87 % of total power usage. The maximum recorded value was 14.24 % in 2020, while the minimum was 0 % in 2000. The fourth independent variable, *a*4, indicates FDI inflows, recorded in Ln terms. The mean Ln FDI inflows are 28.59 billion USD, with a maximum of 31 billion USD in 2020 and a minimum of 24.49 billion USD in 2000. The fifth independent variable, a5, denotes exports, with the mean Ln exports valued at 26.7 billion USD, reaching a maximum of 28.9 billion USD in 2020 and a minimum of 24.66 billion USD in 2000. The sixth independent variable, a6, refers to imports, with a mean Ln import value of 26.68 billion USD. The maximum import value is 28.57 billion USD in 2020, while the minimum is 24.49 billion USD in 2000. The seventh independent variable *a*7 represents GDP, recorded in Ln terms, with a mean Ln GDP of 27.43 billion USD. The maximum GDP is 30.3 billion USD in 2020, and the minimum is 25.22 billion USD in 2000. The eighth independent variable *a*8 reflects population figures, expressed in Ln terms, with a mean Ln population of 17.40334 individuals. The maximum population figure is 21.06763 individuals in 2020, while the minimum is 15.20875 individuals in 2000.

**Fig. 2:**
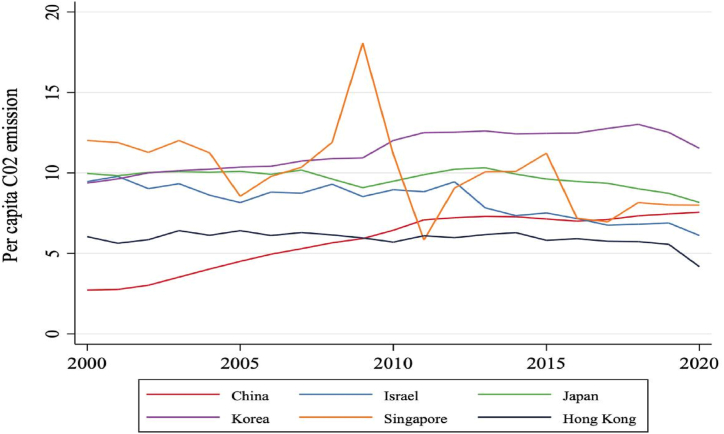
The per capita carbon dioxide output for the six selected countries from 2000 to 2022 (measured in tons). (Source: compiled by the authors).

Fig. 2 displays the per capita carbon dioxide output of the six developed Asian nations. Overall, carbon dioxide output per capita remained relatively stable over the 20 years, experiencing a decline in recent years, which can be attributed to policymakers' adherence to the UN's Sustainable Development Goals (SDGs). This trend indicates a growing concern for ecological preservation among citizens in these countries. Notably, Singapore's carbon dioxide output per capita rose between 2008 and 2010 but decreased in subsequent years. Hong Kong recorded the lowest per capita carbon dioxide output among the six countries, with a reduction observed from 2018.

**Fig. 3:**
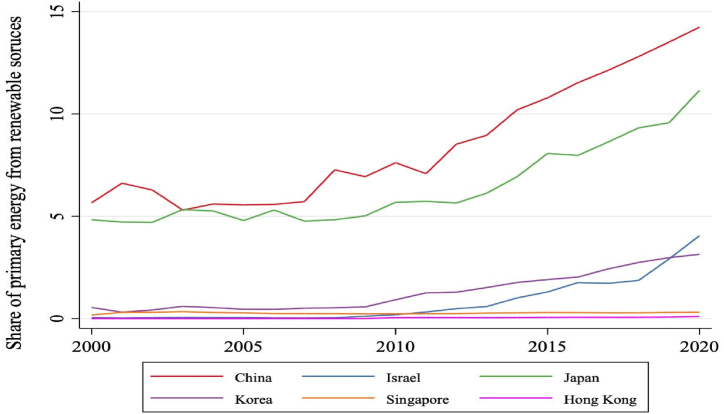
The proportion of total energy consumption accounted for by sustainable energy in the six selected countries from 2000 to 2022 (measured in tons). (Source: compiled by the authors).

In contrast, South Korea has the highest per capita carbon dioxide output, although it has decreased in recent years. China's per capita carbon dioxide output has slightly increased over the past two decades, remaining lower than those of Singapore, Israel, and South Korea. Recent data reveal a carbon dioxide output per capita decline across all six developed Asian countries. Fig. 3 illustrates the percentage of power usage derived from sustainable energy sources from 2000 to 2020. Overall, sustainable energy consumption has risen over the past 20 years. In 2020, China had the highest proportion of sustainable energy consumption at 15 %, while South Korea had the lowest at 3 %.

The findings regarding the relationship between the dependent variable (Q) and the independent variables are displayed in Table 3. The study employs the random effects model and the Cobb-Douglas framework to address the research questions.

**Table 3:** The Cobb-Douglas regression model analyzes the relationship between carbon dioxide output (Q) and power usage, energy from fossil sources, sustainable energy consumption, FDI inflows, exports, imports, GDP, and population for six developed Asian countries from 2000 to 2020.

Random-effects GLS regression	Number of obs	=	124			
Group variable: id	Number of groups	=	6			
R-squared:	Obs per group:					
Within = 0.6632	Min	=	19			
Between = 0.9999	avg	=	20.7			
Overall = 0.9967	max	=	21			
	Wald chi2(8)	=	34365.36			
corr(u_i, X) = 0 (assumed)	Prob > chi2	=	0			
Ln CO_2_ emission	Coefficient	Std. err.	z	P > z	[95 % conf.	interval]
Ln Electricity Consumption	0.8693∗∗∗	0.0726708	11.96	0.000	0.7269	1.0117
Ln Fossil Fuel Energy Consumption	0.0924∗∗	0.0397417	2.32	0.020	0.0145	0.1703
Renewable Energy Consumption	−0.0039∗∗∗	0.0073043	0.53	0.006	−0.0104	0.0182
Ln FDI inflows	−0.0480∗∗∗	0.0161237	−2.98	0.003	−0.0796	−0.0164
Ln Exports	0.0191∗∗∗	0.0393582	0.49	0.006	−0.0580	0.0963
Ln Imports	0.0624∗∗∗	0.0493286	1.26	0.002	−0.0343	0.1591
Ln GDP	−0.2685∗∗∗	0.0471255	−5.7	0.000	−0.3608	−0.1761
Ln Population	1.2483∗∗∗	0.027973	44.62	0.000	1.1934	1.3031
_cons	−18.1196∗∗∗	0.6469466	−28.01	0.000	−19.3876	−16.8516

Note: ∗∗∗P < 0.01 (Sources: complied by authors).

The overall R-squared value of 0.9967 indicates that the variations in the dependent variable can be attributed to approximately 99.67 % of the differences in the independent variables. The statistical significance is demonstrated by the low P-values across multiple variables: power usage (P = 0.000), fossil fuel variables (P = 0.020), sustainable energy consumption (P = 0.006), FDI inflows (P = 0.003), exports of goods and services (P = 0.006), imports of goods and services (P = 0.002), economic development (P = 0.000), and population size (P = 0.000). These results, detailed in equation (12), underscore significant relationships between these factors and carbon dioxide (CO2) emissions in the six selected developed Asian countries.

(12) Ln(Qi,t) = -18.1196 + 0.8693Ln(a1i,t)+0.0924Ln(a2i,t)-0.0039Ln(a3i,t)-0.048Ln(a4i,t)+0.0191Ln(a5i,t)+0.0624Ln(a6i,t)-0.2685Ln(a7i,t)+ 1.2483Ln(a8i,t) + ei,t

The study calculates the elasticity of carbon dioxide output regarding power usage as E = 0.8693. The findings indicate that a 1 % increase in power usage corresponds to a 0.87 % rise in carbon dioxide output. This issue suggests that China, Hong Kong, Singapore, South Korea, Japan, and Israel predominantly rely on electricity from polluting sources, meaning increased electricity usage leads to higher carbon dioxide output. The elasticity of carbon dioxide output concerning fossil fuel consumption is computed as E = 0.924. The results show that a 1 % rise in fossil fuel consumption results in a 0.9 % increase in carbon dioxide output. This finding reinforces that these six countries utilize a significant amount of electricity from environmentally harmful fossil fuels, indicating that carbon dioxide output also increases as fossil fuel consumption rises.

In contrast, the elasticity of carbon dioxide output related to sustainable energy consumption is calculated as E = −0.0039. The results reveal that a 1 % increase in sustainable energy consumption leads to a decrease of 0.00398 % in carbon dioxide output. This issue demonstrates that China, Hong Kong, Singapore, South Korea, Japan, and Israel are actively promoting and utilizing green energy sources to help reduce carbon dioxide output. The study also computes the elasticity of carbon dioxide output concerning cross-border investment (FDI) inflows as E = −0.048. The findings indicate that a 1 % rise in FDI inflows corresponds to a 0.048 % reduction in carbon dioxide output. This issue suggests that these countries are attracting environmentally friendly FDI, which contributes to lowering carbon dioxide output.

Furthermore, the elasticity of carbon dioxide output regarding exports is calculated as E = 0.0191. Findings demonstrate that a 1 % increase in the exports of goods and services leads to a 0.02 % rise in carbon dioxide output, implying that the products exported by these countries have a minor environmental impact. The elasticity related to imports is measured as E = 0.0624, showing that a 1 % increase in imports results in a 0.06 % rise in carbon dioxide output. This issue indicates that imported products also contribute to ecological contamination.

The elasticity of carbon dioxide output in economic development is computed as E = −0.2685. The findings reveal that a 1 % increase in economic development corresponds to a 0.27 % reduction in carbon dioxide output. This issue suggests that China, Hong Kong, Singapore, South Korea, Japan, and Israel emphasize green and long-term environmental growth, as evidenced by smaller increases in carbon dioxide output compared to other economies. Higher economic development is associated with a healthier environment, offering a model for other nations aiming to grow economically while protecting their environment. Finally, the elasticity of carbon dioxide output concerning population growth is calculated as E = 1.2483. Research indicates that a 1 % increase in population is associated with a 1.283 % increase in carbon dioxide output, highlighting that these countries' carbon dioxide output per capita is rising. This trend can be attributed to these nations' relatively low population density, contributing to higher per capita emissions. The subsequent section presents the discussion, conclusions, and recommendations based on these research findings.

## 5 Discussion

Key Findings- The results of this study highlight several vital relationships between power usage, fossil fuel use, sustainable energy adoption, FDI, imports, exports, economic development, population, and ecological contamination in the context of six developed Asian countries. It can be inferred from the results that while economic development and globalization contribute to ecological contamination, shifts toward sustainable energy and stringent environmental policies can mitigate these adverse effects. Electricity Consumption and Environmental Pollution- The analysis confirms that increased power usage drives carbon dioxide output in the studied countries. This finding aligns with existing literature, such as [2,18,19,99], highlighting the environmental costs associated with high energy use. The positive correlation between power usage and pollution underscores the need for energy efficiency measures and the adoption of cleaner technologies to reduce the environmental impact of energy use.

Fossil Fuel Use vs. Renewable Energy- The results clearly distinguish between the environmental impacts of fossil fuel use and sustainable energy adoption. Higher reliance on fossil fuels is associated with increased carbon dioxide output, consistent with the findings of the International Energy Agency (2019). In contrast, adopting sustainable energy sources is linked to lower pollution levels, supporting the conclusions of [4,18,99,100]. These findings emphasize the importance of transitioning from fossil fuels to sustainable energy for sustainable environmental outcomes. Foreign Direct Investment (FDI)- The study finds that FDI inflows are negatively associated with carbon dioxide output, suggesting that increased foreign investment may lead to lower pollution levels. This result partially supports the green FDI in 06 Asian developed countries-hypothesis [1,6], which posits that foreign firms may relocate to countries with less stringent rules protecting the environment. However, the impact of FDI on pollution can vary based on the environmental policies of host countries. Countries with robust rules protecting the environment may attract cleaner technologies and investments, mitigating the adverse effects of FDI on pollution.

Trade Activities: Imports and Exports- The findings indicate that imports and exports positively correlate with carbon dioxide output, though the significant levels vary. This result aligns with the scale effect described by Refs. [7,101], where increased trade volumes lead to higher economic activity and, consequently, more significant pollution. However, the technique effect, which suggests that trade can promote cleaner technologies and environmental standards, needs to be more pronounced in the context of the studied countries. The composition of traded goods likely plays a critical role in determining the net environmental impact of trade activities. Economic growth in 06 Asian developed countries is the green economic development. Therefore, the greater the economic development, the lower the ecological contamination. Population-population size is a significant driver of ecological contamination. Larger populations are associated with increased carbon dioxide output, consistent with the Environmental Kuznets Curve (EKC) hypothesis posited by Refs. [9,[102], [103], [104]]. However, the findings also highlight the importance of sustainable economic practices and demographic policies in mitigating the environmental impacts of growth and population dynamics. The negative relationship between economic development and pollution suggests that economic development may decrease environmental degradation with appropriate rules protecting the environment and investments in cleaner technologies.

Policy Implications- The results of this study have several policy implications for the six developed Asian countries: Energy Efficiency and Clean Technologies: Governments should prioritize energy efficiency measures and the adoption of clean technologies to reduce the environmental impact of power usage. Incentives for energy-efficient practices and investments in sustainable energy infrastructure can help mitigate pollution. Transition to Renewable Energy: Policymakers should accelerate the transition from fossil fuels to sustainable energy sources. Implementing policies that support sustainable energy adoption, such as subsidies, tax incentives, and regulatory frameworks, can significantly reduce carbon dioxide output. Environmental Regulations for FDI: the study has policies to mitigate the potential negative impacts of FDI on the environment, and countries should enforce stringent rules protecting the environment. Attracting green investments and promoting technologies that reduce pollution can enhance the positive effects of FDI. Sustainable Trade Practices: Governments should consider the environmental implications of trade policies. Encouraging the trade of environmentally friendly products and implementing regulations to control pollution-intensive industries can help balance trade activities with ecological sustainability. Integrated Economic and Environmental Policies: The positive relationship between economic development, population, and pollution underscores the need for integrated policies that promote long-term environmental growth. Investments in clean technologies, rules protecting the environment, and sustainable economic practices are crucial for reducing the environmental impact of growth.

Understanding the intricate dynamics between economic activities, energy consumption, and ecological contamination is paramount for long-term environmental growth, especially in developed Asian countries. This study investigates these relationships from 2000 to 2020 across six nations: Japan, South Korea, Singapore, Hong Kong, China, and Israel. The selection of variables—power usage, fossil fuel use, sustainable energy adoption, cross-border investment (FDI), trade dynamics (imports and exports), economic development, population dynamics, and carbon dioxide output—is guided by established theoretical frameworks and informed by prior literature. This section critically reviews relevant theories, justifies variable choices, and underscores the study's innovative contributions, particularly in exploring the role of digital technology in shaping future research directions. Theoretical Foundations- Environmental Kuznets Curve (EKC): The Environmental Kuznets Curve posits a nonlinear relationship between economic development and environmental degradation. This theory suggests that environmental quality initially deteriorates with economic development but improves after reaching a certain income level as societies prioritize environmental conservation and adopt cleaner technologies [10,99].

Energy Transition Theory- Energy transition theory emphasizes the shift from fossil fuels to sustainable energy sources as a pivotal strategy for mitigating environmental impacts. It underscores the role of policy interventions and technological advancements in accelerating the adoption of sustainable energy solutions [105]. Justification of Variables: Electricity Consumption (ELEC)- Electricity consumption is a proxy for economic activity and energy demand. Higher electricity usage is typically associated with industrial production and urbanization, contributing to increased carbon dioxide output if sourced from fossil fuels [106]. Fossil Fuel Use (FFUEL) and Renewable Energy Adoption (RENE): Choosing between fossil fuels and sustainable energy sources significantly influences environmental outcomes. Fossil fuel dependency exacerbates carbon emissions, whereas sustainable energy adoption offers cleaner alternatives to mitigate environmental impacts [12,106].

Foreign Direct Investment (FDI): FDI plays a dual role in economic development and environmental management. While it promotes technological transfer and industrialization, unregulated FDI may lead to environmental degradation without stringent safeguards [107]. Trade Dynamics (IM and EX). International trade affects ecological sustainability by influencing production patterns and resource consumption. Increased trade volumes can amplify environmental pressures through transportation emissions and industrial activities [108]. Economic Growth (GDP) and Population (POP): Economic Growth and population dynamics drive energy consumption patterns and resource utilization. Rapid economic development and population growth pose challenges to ecological sustainability, necessitating innovative policy responses [4,6,7,109].

Contribution to Literature- This study contributes to the literature in several key ways: Integrated Analysis: Integrates multiple variables within a robust analytical model to comprehensively understand ecological contamination dynamics in developed Asian economies. Empirical Rigor: Applies advanced panel data regression techniques to quantitatively analyze the relationships between economic activities, energy consumption, and carbon dioxide output, enhancing methodological reliability. Policy Implications: Offers supported by empirical evidence insights for policymakers to formulate effective long-term environmental growth and environmental management strategies tailored to the specific challenges and opportunities in developed Asian countries. Role of Digital Technology in Future Research: Looking ahead, digital technology plays a transformative role in shaping future research directions. Data Integration and Analytics: Advances in data analytics enable researchers to analyze vast datasets and extract meaningful insights into complex environmental systems, enhancing the precision and scope of empirical studies. Simulation and Modeling: Digital simulations and modelling techniques facilitate scenario analysis, allowing researchers to predict the impacts of policy interventions on environmental outcomes with greater accuracy and foresight.

Innovation and Collaboration: Digital platforms foster global collaboration among researchers, policymakers, and industry stakeholders, facilitating knowledge exchange and accelerating the adoption of sustainable technologies and practices. Relevance and Future Directions- By embracing digital technology, future research can deepen our understanding of the dynamic interactions between economic development, energy consumption, and ecological sustainability. This collaborative approach not only builds on the findings of this study but also expands the frontiers of knowledge, offering innovative solutions to global environmental challenges. Ultimately, integrating digital tools into research methodologies empowers stakeholders to make informed decisions and advance toward a more sustainable future for all.

## 6 Conclusions and recommendations

This study contributes significant insights into the relationships between economic activities, energy consumption patterns, and ecological contamination across six developed Asian countries from 2000 to 2020. By employing robust panel data regression techniques and analyzing a comprehensive set of variables, including power usage, fossil fuel use, sustainable energy adoption, cross-border investment (FDI), trade dynamics, economic development, and population, the research provides nuanced findings that advance the understanding of sustainability challenges in this region. Scientific Value-Added- The study's methodology builds upon established research approaches while addressing critical gaps in the literature. Empirical Rigor: The paper utilizes both fixed-effects and random-effects models, and the study controls for country-specific effects and temporal variations, ensuring robustness in the analysis of complex interactions among variables.

Comparative Analysis: By comparing findings with benchmarks from previous studies, the research quantitatively assesses the impact of economic policies and energy transitions on carbon dioxide output in developed Asian economies. Applicability of Findings- The findings directly affect policymakers and stakeholders- Policy Recommendations: The study identifies pathways for reducing carbon dioxide output through enhanced energy efficiency measures, accelerated adoption of sustainable energy sources, and targeted environmental policies. These recommendations are crucial for achieving long-term environmental growth goals and meeting international climate commitments. Regional Context: By focusing on developed Asian countries, the research offers insights tailored to these nations' specific socio-economic and environmental challenges. This context-specific approach enhances the relevance and applicability of the findings for policy formulation and implementation.

Novelty and Significance- New Insights: The study uncovers novel insights into the dynamics between economic development, energy consumption, and ecological sustainability in developed Asian countries. It highlights how energy policy and economic activity variations influence carbon dioxide output differently across countries and over time. Quantitative Comparison: Quantitative reasoning and benchmark comparisons with previous studies underscore the novelty of the findings. For instance, the study quantifies the relative impact of sustainable energy adoption versus fossil fuel use on carbon dioxide output, providing clear benchmarks for policy prioritization. Future Directions- Future research could expand on this study by Longitudinal Analysis: Investigating more extended periods to capture evolving trends and policy impacts on environmental outcomes. Policy Simulation: Conduct scenario analyses to project the potential effects of alternative policy interventions on carbon dioxide output and environmental quality. In conclusion, this study advances scientific understanding and offers practical insights into addressing environmental challenges in developed Asian economies. By integrating empirical evidence with policy implications, the research contributes to informed decision-making towards achieving long-term environmental growth goals and enhancing environmental stewardship globally.

Electricity Consumption and Economic Growth: As nations progress and industrialize, they generally require increased energy to sustain their economies. This issue often results in higher power usage, particularly in energy-intensive manufacturing and transportation sectors. However, this relationship is only sometimes straightforward, as some countries may focus on improving energy efficiency and investing in sustainable energy to lessen their reliance on fossil fuels and address climate change. Using fossil fuel sources, such as coal, gasoline, and oil, contributes to CO2 output in the atmosphere, intensifying global warming and climate change. Countries that predominantly generate energy from fossil fuels tend to experience higher greenhouse gas emissions than those prioritizing sustainable energy and energy-efficient practices. Renewable Energy Consumption and Greenhouse Gas Emissions: Renewable energy sources, such as solar, wind, and hydropower, produce electricity without emitting greenhouse gases. Nations investing in developing sustainable energy typically have lower greenhouse gas emissions than those heavily dependent on fossil fuels. The findings indicate that the six selected developed Asian countries have effectively utilized sustainable energy to protect the environment and reduce carbon dioxide output.

FDI Inflows and Greenhouse Gas Emissions: Foreign direct investment (FDI) can bring capital, technology, and expertise to a country. However, if FDI is directed toward sectors that rely significantly on fossil fuels, it may also increase energy consumption and greenhouse gas emissions. The research results prove that the six selected developed Asian countries attracted green and sustainable FDI flows. Therefore, the greater the FDI inflows in these countries, the better the environment. The imports of goods and exports and energy consumption-the imports and exports of goods and services can affect a country's energy consumption and greenhouse gas emissions, mainly if the imported or exported goods are energy-intensive or produced using fossil fuels. Countries prioritizing sustainable trade policies and investing in low-carbon technologies will likely have lower environmental impacts from trade. The research results prove that the six selected developed Asian countries imported and exported goods and services that pollute the environment.

Population Growth and Energy Consumption: As populations increase, the need for energy to power homes, businesses, and transportation also rises. Nations that focus on long-term environmental growth and invest in sustainable energy, energy efficiency, and low-carbon technologies are typically better prepared to meet this growing demand while minimizing their environmental footprint. Overall, the relationships among power usage, energy from fossil sources, sustainable energy consumption, cross-border investment (FDI) inflows, imports and exports of goods and services, economic development, population dynamics, and carbon dioxide output are intricate and interrelated. Countries prioritizing long-term environmental growth and investing in sustainable energy and energy-efficient practices will likely experience long-term economic and environmental advantages.

## 7 Limitations and future research directions

This study recognizes several limitations. Firstly, the availability and quality of data may differ across countries and periods, which could influence the reliability of the findings. Secondly, the research is confined to six developed Asian nations, potentially limiting the applicability of the results to other regions. Thirdly, there may be endogeneity concerns stemming from the interconnections among the variables that this study needs to address fully. Future research could broaden its scope by including additional countries and extending the timeframe for a more thorough analysis.

Moreover, employing advanced econometric methods, such as instrumental variable techniques or dynamic panel data models, could help tackle potential endogeneity issues and yield more robust insights. Exploring the effects of specific environmental policies and regulations on the relationship between economic activities and pollution could also provide valuable strategic suggestions for promoting long-term environmental growth. This paper has limitations, such as not investigating the relationship between forested areas and innovation in reducing carbon dioxide output, as explored by Balsalobre et al., Khan et al., and Raihan et al. The authors intend to address these topics in future research within these countries.

## CRediT authorship contribution statement

**Pham Xuan Hoa:** Writing – review & editing, Writing – original draft, Visualization, Validation, Supervision, Software, Funding acquisition, Formal analysis, Data curation. **Vu Ngoc Xuan:** Project administration, Methodology, Investigation, Funding acquisition, Formal analysis, Data curation, Conceptualization. **Nguyen Thi Phuong Thu:** Software, Resources, Project administration, Methodology, Investigation.

## Data availability statement

The data that has been used is confidential.

## Declaration of competing interest

The authors declare the following financial interests/personal relationships which may be considered as potential competing interests:Vu Ngoc Xuan reports financial support was provided by 10.13039/501100019220National Economics University. Vu Ngoc Xuan reports a relationship with National economics university that includes: employment. If there are other authors, they declare that they have no known competing financial interests or personal relationships that could have appeared to influence the work reported in this paper.
